# Recent advances in acute gastrointestinal graft versus host disease (aGvHD): aspects of steroid-resistant disease

**DOI:** 10.1007/s00277-024-05952-0

**Published:** 2024-08-29

**Authors:** Joanna Kujawska, Robert Zeiser, Lidia Gil

**Affiliations:** 1 Department of Hematology and Bone Marrow Transplantation, Poznan University of Medical Sciences, Poznań, Poland; 2 Department of Internal Medicine I, Faculty of Medicine, Medical Center-University of Freiburg, University of Freiburg, Freiburg, Germany

**Keywords:** Hematopoietic stem cell transplantation (HSCT), Graft versus host disease (GvHD), Steroid-resistant GvHD (SR-GvHD), Gut microbiota

## Abstract

Acute Graft versus Host Disease (aGvHD) is a common immunological complication occurring in patients undergoing allogeneic hematopoietic stem cell transplantation (allo-HSCT). Moreover, aGvHD is associated with a higher risk of infections and metabolic complications, affecting non-relapse mortality. Progress in transplantation has changed the prophylactic and therapeutic strategies of aGvHD and improved patient outcomes. The standard first-line therapy remains steroids, with a response rate of about 50%. The Janus Kinase 2 (JAK2) inhibitor, ruxolitinib, is an effective second-line therapy. The management of patients who developed a disease that is refractory to steroids and ruxolitinib, especially in the severe gastrointestinal forms of aGvHD, is not validated and remains an unmet medical need. In the article, we present the current clinical practice, as well as the latest advances targeting pathophysiological pathways of GvHD and gut microbiota, which may be a potential future of aGvHD therapy.

## Introduction

Allogeneic hematopoietic stem cell transplantation (allo-HSCT) is a well-established curative therapy for various malignant and non-malignant hematological diseases [1]. Despite the continuous optimization of the allo-HSCT procedure, the success of allo-HSCT is limited by potentially life-threatening complications, including graft versus host disease (GvHD). GvHD occurs when immune-competent donor cells recognize the patient’s cells as foreign. The immune response directed against the patient’s major histocompatibility complex (MHC) and/or minor histocompatibility antigen (miH) disparities that are expressed by various cells i.e. antigen-presenting cells (APCs), patient’s B-cells, dendritic cells (DC), macrophages and monocytes, activates donor T cells, which, in the cytolytic mechanism, eliminate the patient’s human leukocyte antigen (HLA) disparate cells inducing acute GvHD. As it may induce durable remission, the immunologic graft versus leukemia effect is wanted when donor T-cells attack abnormal cells. However, the immunological mechanism of GvHD refers to malignant cells and other healthy recipient tissues, resulting in a spectrum of clinical manifestations ranging from mild skin involvement to severe systemic organ dysfunction [2]. The mouse models of acute GvHD pathophysiology help us to understand the complex biology of acute GvHD and find the potential pathway for targeted therapeutic intervention. Recent data show that pro- and anti-inflammatory neutrophils contribute or inhibit intestinal GvHD [3, 4].

According to the National Institutes of Health (NIH) classification, a classic acute graft versus host disease (aGvHD) occurs within 100 days after allo-HSCT. It affects the skin, liver, and gastrointestinal (GI) tract. However, depending on the timing of the clinical manifestations, we can distinguish late onset aGvHD (the first episode occurs later than + 100d), recurrent aGvHD (the new episode in a patient with a medical history of classic aGvHD), and persistent aGvHD (the classic manifestation persisting more than + 100d). Chronic GvHD (cGvHD) may affect every organ without any time limit. When the typical symptoms of aGvHD persist with signs of cGvHD, it is classified as an overlapping syndrome [5–7]. The diagnosis of aGvHD is based on clinical symptoms and requires detailed differential diagnoses. The Mount Sinai Acute GVHD International Consortium (MAGIC) created a standardized, expert consensus of aGvHD diagnosis and clinical staging [8].

Over the years, the incidence and mortality of aGvHD have decreased [9]. AGvHD, at any severity, may affect up to 40–60% of allo-HSCT recipients [5, 10]. Clinically significant grades 2 to 4 of aGvHD, despite prophylaxis, remain challenging as one of the leading causes of non-relapse mortality (NRM) in patients undergoing allo-HSCT [11]. The CIBMTR data from transplant centers in the US present that in the early posttransplant period (within 100 days after alloHSCT), GvHD is the third cause of death unrelated to relapse (15%), after infection (25%) and organ failure (24%) [12]. About 50% of aGVHD patients respond to the standard first-line therapy with systemic, high-dose glucocorticoids (GC) [13], but the response rate in patients with grade 4 disease does not exceed 30% [14]. The outcome of steroid-resistant aGvHD (SR-aGvHD) is poor [6, 13, 15], especially in patients with the dominant gastrointestinal form (GI-aGvHD), who are vulnerable to life-threatening complications such as infections, bleeding, and surgical complications [16]. According to real-life data, the percentage of SR-aGvHD is higher in patients with GI-GvHD, and the median survival rate is less than 12 months [17]. The clinical approach for SR-aGvHD differs across centers. Ruxolitinib was approved for SR-aGvHD patients by the FDA and EMA in 2019 [18] based on promising results from the REACH2 study [19] and has become a standard second-line therapy in SR-aGvHD [6, 20]. The access to ruxolitinib and local experience with the therapy vary by country, e.g., public financing and broader access to ruxolitinib treatment in SR-aGvHD in Poland started in September 2023 [21].

The recent advances in prophylaxis and treatment of aGvHD improve patient outcomes. However, the median survival in individuals who did not respond to the first- and second-line treatment is still low. The paper discusses current clinical approaches and supportive care. In particular, we are considering patients with GI SR-aGvHD.

### Standard prophylaxis

The recently updated recommendations by the European Society for Blood and Marrow Transplantation (EBMT) present the consensus of clinical practice and prioritize research areas needing further investigation in prophylaxis and treatment of aGvHD. The decision on a prophylaxis regimen depends on several factors: donor type, degree of human leukocyte antigen match, conditioning intensity, and general condition of a patient [13, 20]. The traditional GvHD prophylactic regimens include a calcineurin inhibitor (CNI) - tacrolimus (TAC), or cyclosporine (CSA) combined with antimetabolites, such as methotrexate (MTX) or mycophenolate mofetil (MMF); with or without antithymocyte globulin (ATG). Standard prophylaxis has shown efficacy; however, it might be associated with significant toxicity, and there is a need to explore advancements in GvHD prophylaxis and improve clinical practice. An overview of the latest reports on GvHD prevention is beyond this review. Still, it is worth mentioning that recently, the use of posttransplant cyclophosphamide (PTCy) in prophylaxis has been increasing [12, 20]. Standard of GvHD prophylaxis in haploidentical transplantation [22] - PTCy is widely used in mismatched unrelated donor (MMUD) HSCT and is gaining importance in matched related (MRD) and unrelated donor (MUD) transplantations. According to EBMT recommendations, the experts do not recommend PTCy for MRD HSCT as preferable GvHD prophylaxis compared to rabbit anti-T-cell globulin (rATG). For GvHD prophylaxis in alloHSCT from MUD and MMUD, the use of rATG or PTCy is preferred to GvHD prevention with neither rATG nor PTCy [20]. Modified GvHD prevention regimens and a different toxicity profile of prophylaxis may have an impact on treatment algorithms for aGvHD in the future.

### Biomarkers – risk stratification, predictive factors

Detecting biomarkers for the aGvHD diagnosis has been a zone of interest for researchers worldwide, and there has been significant progress in improving knowledge about aGvHD pathogenesis. Proteins that are pro-inflammatory cytokines and factors related to GvHD-specific organ damage measured at defined time points have been evaluated as potential biomarkers. Plasma levels of elafin increase at the onset of skin aGvHD [23]; regenerating islet-derived protein 3α (REG3A), cytokeratin 18 (CK18), and hepatocyte growth factor levels are significantly higher in patients with lower GI acute GVHD compared to patients suffering from non-GvHD diarrhea [24, 25], these last three biomarkers increase in patients with liver aGvHD; however, they do not distinguish other potential causes of hyperbilirubinemia [26]. The elevated levels of the ST2 biomarker (suppression tumorigenicity 2) are associated with the risk of severe aGvHD and improved risk stratification for response to first-line therapy in patients with aGvHD. The biomarkers panel evaluated, i.e., by the MAGIC consortium, may have the greatest clinical significance and can be used to stratify the risk of aGvHD and predict the outcome [8]. In high-risk patients with GI-aGvHD, predictive models using biomarkers can be helpful for clinicians but have yet to be widely used in everyday clinical practice [27].

### First-line therapy – new perspectives

According to EBMT recommendations, in most cases, the skin-limited clinical manifestation without organ involvement (grade 1) requires topical steroids alone. The decision of systemic treatment for aGvHD is based on clinical signs and should be introduced in patients with aGvHD grade 2 or higher. The standard first-line therapy is methylprednisolone with an initial dose of 2 mg/kg per day or prednisone equivalent [13]. In GI-aGvHD, adding non-absorbable oral steroids such as budesonide (9 mg/d) or beclomethasone (1.3 mg-2.0 mg four times daily) is recommended [28, 29].

Assessment of aGvHD should be performed 3–7 days after diagnosis and introduction of first-line therapy. Failure of steroid treatment is defined as the progression of aGvHD symptoms by day three or lack of improvement by day 7 [14]. A short-term criterion of response in skin and liver involvement might be appropriate. However, from a practical standpoint, starting tapering steroids might be an issue in GI-aGvHD patients due to the requirement of epithelial repair to reduce stool volume, which is involved in MAGIC response criteria [1, 2]. Decreased stool volume should coexist with the increasing ability of oral intake. Therefore, considering the severity of symptoms, waiting 14 days should be considered before therapy modification.

Prolonged, high-dose systemic steroids remain the initial therapy for acute graft-vs.-host disease. As the response rates to first-line treatment with steroids could be better (~ 50%) [9, 13, 14], strategies to improve response and minimize steroid exposure are needed. Long-term use of steroids may lead to complications such as electrolyte disturbances, neuropathy, osteoporosis, and therapy-induced diabetes [6, 13]. A significantly higher risk of infection, including cytomegalovirus (CMV) reactivation and invasive fungal infections in transplant recipients, requires close monitoring, prophylaxis, and immediate therapy of suspected infection [6, 30, 31]. Therefore, there is an unmet need for improvement in the treatment strategy by predicting response after first-line treatment and adding other therapies to improve response rates.

A steroid-free first-line strategy might be evaluated among patients under protocols with GvHD prophylaxis without calcineurin inhibitors (CNI), i.e., posttransplant cyclophosphamide or αβ ex vivo T-cell depletion. In a recently published study in patients undergoing allo-HSCT with these prophylaxis protocols (34 patients with grade II-IV aGvHD and 40 with moderate and severe cGVHD), CNIs or other steroid-free regimens were administered as the first-line. ORR was significantly higher in cGVHD than in aGVHD: 80% (95% CI 68–92) vs. 47% (95% CI 30–64%), *p* = 0.0031. Responders had lower use of systemic anti-infective therapies [32]. The steroid-free approach is somewhat controversial, as data from randomized studies are lacking; however, in the future, in patients with contraindications to use prolonged, high-dose steroids, it might be worth evaluating in further studies.

For many years, there were trials in newly diagnosed patients evaluating the addition of molecules related to the pathogenesis of aGvHD to improve response rates. Although some of the agents might be efficient in the therapy of SR-aGvHD, combined with standard first-line treatment, in most cases, was not beneficial compared to corticosteroids alone. Infliximab, mycophenolate mofetil (MMF), etanercept, denileukin diftitox, pentostatin, or natalizumab are examples of molecules that were assessed and did not prove efficacy to become a key player in combined first-line aGvHD therapy [33–37].

As ruxolitinib - a selective Janus kinase (JAK) 1/2 inhibitor, has been approved for the therapy of SR-aGvHD and is well tolerated, there are ideas of adding ruxolitinib to steroids to improve the efficacy of the first-line. An open-label, multicenter, prospective, randomized phase II trial compares a two-arm combination of ruxolitinib with a lower dose of methylprednisolone (1 mg/kg) and ciclosporin (CSA) versus a standard dose of methylprednisolone (2 mg/kg) with CSA in 198 patients newly diagnosed with moderate- to severe-risk aGvHD [8, 38]. The clinical trial was registered in July 2019 with ClinicalTrials.gov NCT04061876, and the final results have not been published yet. Initial data on the subgroup from the study suggest that in all aGvHD patients treated with steroids-ruxolitinib therapy, the CR rate at day 28 was 82%, and in patients with GI-aGvHD was 84% (21/25). Interestingly, the kinetics of MAGIC biomarkers were different in the experimental group. The data suggest that the concentration of REG3α may predict refractory aGvHD in patients treated with combined therapy with steroids and ruxolitinib, which still needs confirmation and further investigation [39]. The EBMT experts emphasize the need for evaluating steroid-free treatment as first-line therapies of aGvHD, which potentially might be successful [20].

The results of a randomized phase II trial of prednisone with or without extracorporeal photopheresis (ECP) as a first-line therapy for patients with aGvHD seem promising, however not groundbreaking in patients with GI-aGvHD [40]. The ECP arm had a higher probability of success (0.815). It was potentially more effective than the arm with steroids alone in aGvHD limited to the skin (response rate: 72% vs. 57%, respectively) than in patients with visceral involvement of aGvHD (47% vs. 43%, respectively). The addition of ECP to steroids may result in higher GvHD response for patients with skin-limited aGvHD; in more severe forms, more data are lacking.

Another selective inhibitor of Janus kinase (JAK)1 – itacitinib - was tested in clinical trials after proving promising efficacy in preclinical models of aGvHD. In the first registered study (NCT02614612), an open-label phase 1 among patients with steroid-naive or steroid-refractory aGvHD, the results of the use of itacitinib with steroids were promising. ORR at day 28 was 75.0% for patients with steroid-naive aGVHD and 70.6% for those with SR aGVHD. In all patients receiving itacitinib, the steroid dose decreased over time [41]. Another study compared corticosteroids with itacitinib versus corticosteroids with a placebo in first-line therapy of aGvHD. In this international, double-blind, adaptive phase 3 study – GRAVITAS-301, registered with ClinicalTrials.gov (NCT03139604), 439 patients with grades II-IV aGVHD were randomized. The ORR at day 28 for itacitinib was 74% (CR 53%) and for placebo 66% (CR 40%). Adding itacitinib to standard first-line therapy did not demonstrate a difference in ORR at day 28 (OR 1.45, 95% CI 0.96–2.20; *p* = 0.078) [42]. More recently, in a multicenter, phase 2 study registered with ClinicalTrials.gov (NCT03846479), investigators hypothesized that itacitinib without steroids might be effective for Low-risk GvHD (LR GvHD) patients, defined based on validated biomarkers and clinical criteria. Itacitinib was administered to 70 patients with LR GvHD in 28 days cycle; when they responded, the cycle was repeated. Those patients were compared to a control group (*n* = 140) treated with standard first-line therapy. The response rate within 7 days was higher in patients treated with itacitinib (81% vs. 66%, *P* = 0.02). ORR at 28 day was high for either the experimental or control group (89% vs. 86%, *P* = 0.67) [43].

Sirolimus – mTOR kinase inhibitor was compared to prednisone as an upfront therapy for patients with standard risk aGvHD (according to Minnesota criteria and Ann Arbor biomarker score [25, 44]). In the multicenter, open-label, randomized phase 2 study, registered at www.clinicaltrials.gov as NCT02806947, 127 patients were randomized (1:1) into groups treated with sirolimus and standard prednisone dose. The response rates on day 28 were similar for both agents (CR/PR; sirolimus: 64.8% (90% CI, 54.1-75.5%) vs. prednisone 73% (90% CI, 63.8-82.2%). The comparison of CR/PR on day 28 with prednisone dose ≤ 0.25 mg/kg/day was favorable for sirolimus (66.7% vs. 31.7%; *P* < 0.001). Steroid exposure, hyperglycemia, and infection grade were reduced in the group treated with sirolimus. However, patients presented an increased risk of thrombotic microangiopathy. The long-term survival outcome was comparable in both groups, and therapy with sirolimus was associated with improved patient-reported quality of life [45]. Further randomized phase 3 study is needed to confirm if sirolimus could be an alternative therapy for patients with standard risk aGvHD who do not tolerate steroids.

GvHD pathogenesis includes complement components C3 and C5. The safety and efficacy of ALXN1007, a monoclonal antibody against C5a, was evaluated in a phase 2a study, registered at www.clinicaltrials.gov as NCT02245412. In twenty-five patients newly diagnosed with low gastrointestinal aGVHD, ALXN1007 was administered with corticosteroids. On day 28, OR was 58% (CR 54%) and 63% by day 56 (all complete responses). There was no correlation between GvHD severity or responses, baseline complement levels (except for C5), activity, or inhibition of C5a with ALXN1007. Promising results require further studies to evaluate the potential role of complement inhibition in aGVHD therapy [46].

Experimental models demonstrated that interleukin-22 is involved in promoting epithelial regeneration and induction of innate antimicrobial molecules [47, 48]. In a multicenter single-arm phase 2 study, registered at www.clinicaltrials.gov as NCT02406651, the safety, and efficacy of F-652, a novel recombinant human interleukin-22 (IL-22), combined with systemic corticosteroids in the initial therapy of 27 patients diagnosed with lower gastrointestinal aGVHD was tested. Treatment response on day 28 was achieved in 19 of 27 patients (70%; 80% confidence interval, 56-79%) [49]. The investigators analyzed a fecal microbiota composition characterized by the increase of commensal anaerobes population in responders, correlating with the protection of microbial diversity. A tissue-supportive approach boosting the recovery of damaged gastrointestinal mucosa and regulating GvHD-associated dysbiosis, especially fecal microbiota transplantation, combined with classical immunosuppressive strategies, can likely be crucial in further studies [50].

After itolizumab, a humanized anti-CD6 mAb demonstrated clinical efficacy in preclinical models of GvHD [51], preliminary data from study Phase 1b/2 with overall response rate ORR at day 29 80% and sustained response rate at day 57 were encouraging [52]. A multi-center study, phase III, compares corticosteroids combined with itolizumab or placebo in the first-line therapy for patients with grade II low GI-aGvHD or Grade III-IV aGVHD. The study is registered at ClinicalTrials.gov ID NCT05263999.

### Steroid resistant GvHD – ruxolitinib

Steroid-refractory/resistant aGvHD is defined as (1) progressive disease after 3 days of corticosteroid therapy (MP 2 mg/kg per day or equivalent); (2) no improvement after at least 7 days of standard first-line therapy; (3) progression to another organ after therapy with a lower dose of steroids (skin limited manifestation or upper gastrointestinal GvHD); (4) recurrence during/after tapering of steroids [14, 53]. The 5-year overall survival in patients with SR-aGvHD is significantly lower than in steroid-sensitive patients, 34.8 vs. 53.3%, *p* = 0.0014. Moreover, steroid resistance is associated with higher 12 months of non-relapse mortality (NRM) when compared to steroid responders (14.3% vs. 39.2%, *p* = 0.016) [17].

As mentioned, no new agents have been recently approved for first- or second-line therapy of aGvHD patients except for ruxolitinib. Due to Janus kinase inhibition, ruxolitinib regulates immune-cell activation and inflammatory cytokines, which cause damage in tissues associated with the pathogenesis of aGvHD [54]. In the open-label, single-group, multicenter study (REACH1), ruxolitinib was used in corticosteroid-refractory patients (grade II-IV aGvHD). At day 28, 54.9% of patients had a response, and 27% of patients presented CR [18, 55, 56]. The results of the REACH2 study proved the initial data. The randomized phase 3 trial compared ruxolitinib with the best available therapy by choice of physician in patients with SR-aGvHD. The overall response rate at day 28 was higher in patients treated with ruxolitinib vs. the control group (62% vs. 39%; odds ratio, 2.64; 95% CI, 1.65–4.22; P,0.001). Moreover, the durable ORR at day 56 was higher in the ruxolitinib than in the control group (40% vs. 22%; odds ratio, 2.38; 95% CI, 1.43–3.94; P 0.001) [19]. In the systematic review, 19 studies were analyzed that presented the response rate of 34 patients with GI-aGvHD ORR: 79.3% (61.4–90.2) [57].

Real-world data presenting a second-line approach in patients with GI-aGvHD from the Transplant Center in Germany indicate that ruxolitinib was the most commonly used therapy, with the overall response at day 28–44.4% and CR 13%, respectively [17]. The real-world data from other countries, i.e., Poland, are limited, where ruxolitinib has been financed since 2023. Evaluation of ruxolitinib efficacy in a Polish single-center experience, after a median of 3 lines of therapies, provides an overall response of 28% of patients with CR in 4 patients and a partial response of 1 patient. One-year overall survival was 60% in ruxolitinib responders versus 31% in patients who did not respond. Unfortunately, 78% of patients died due to GvHD progression [58].

Experts’ consensus defined ruxolitinib-refractory aGvHD as 1) progression comparing baseline after at least 5 to 10 days of ruxolitinib therapy (including an increase in stage/grade or involvement of new organ; 2) persistent disease without partial response or better after ‡14 days of treatment; 3) loss of response – worsening of GvHD symptoms after initial improvement [14]. According to retrospective analysis, ruxolitinib resistance or intolerance occurs in 1/5 of patients with SR-aGvHD [59]. The findings show that the response of SR-GVHD patients is low, and novel approaches, especially in groups of GI-SR-aGvHD, should be explored.

### Steroid and ruxolitinib refractory aGvHD – new directions

Vedolizumab, a monoclonal humanized antibody targeting the α4β7 integrin receptors in guts, prevents the trafficking of T lymphocytes homing the gastrointestinal tract using blockage of T helpers cells and T regulatory cells residing in the colon, has been previously evaluated in patients with inflammatory bowel diseases. Considering the pathophysiology of GvHD, it might be efficient in patients with gastrointestinal involvement.

In several retrospective studies, vedolizumab proved to have encouraging outcomes in patients with SR-GI-aGvHD. Mehta et al. reported that the response at 28-day ORR was 35% (7/20; CR 20%); in ruxolitinib-resistant patients, the response was 50% (6/12; CR 25%), and the 6-month OS was 35% (95% CI 16–55) [60]. In another multicenter study, at 6 to 10 weeks after the beginning of vedolizumab therapy, the ORR was 64%, and the reported 6-month OS was 54% [61]. Polish data report successful cases in the pediatric population where vedolizumab was used as salvage therapy [62]. According to the systematic review, vedolizumab was well tolerated and presented potential effectiveness even in ruxolitinib-resistant patients for SR-LGI aGVHD. However, prospective studies are needed [63].

### Fecal microbiota transplantation

The immunological system of the gut is the first line of protection against ingested microorganisms and other substances. The defense is directed against potentially pathological germs. The tolerance of beneficial commensal bacteria, living symbiotically with our microbiota, is crucial for the intestinal immune system. In patients undergoing HSCT, the intestinal microbiota is damaged due to the conditioning regimens, antibiotics used concurrently with the chemotherapy, and nutritional changes. Studies have proven the loss of microbiota diversity in those patients and found a decrease in commensal types of bacteria for an increase in potentially pathological strains of bacteria, including *Enterococcus* and *Escherichia* species [64–66].

Loss of microbiota diversity is considered a predictive factor of HSCT outcome and steroid-refractoriness in patients with GI-aGvHD [64, 67–69]. The strategies of microbiota recovery in HSCT patients are limited due to prolonged hospitalization, nutritional restriction, and, most importantly, neutropenia occurring in the posttransplant period. Nowadays, fecal microbiota transplantation (FMT) is a well-established method for recurrent Clostridioides difficile infection (CDI), with effectiveness reaching even 90% [70]. The safety and efficacy of FMT in patients with SR-GI-GvHD are clinically relevant. According to preliminary data from case reports and pilot studies [71–73], the ORR of FMT in GI-aGvHD could exceed 70%, with CR near 50% [74]. Polish group reported cases of combined therapy with FMT and ruxolitinib with encouraging results [75]. A clinical trial with the same combination was used as a salvage therapy in 21 patients with SR-aGvHD (registered as NCT03148743). On 28 day, the ORR was 71.4% (95% CI 50.4–92.5%), and nearly half of patients achieved CR [76]. Promising initial results of the combination should be verified in prospective, randomized studies. In the context of immunocompromised patients, rotes of FMT administration should be carefully selected. FMT can be delivered through the upper gut (oral intake, nasogastric tube, esophagogastroduodenoscopy, enteroscopy), midgut (nasojejunal/enteral tube, jejunostomy, or percutaneous endoscopic cecostomy), or lower gut (enteroscopy, transendoscopic enteral tube, enema, and colonoscopy). According to gastroenterological recommendations for FMT in recurrent CDI, there is no insufficient evidence to recommend a specific route of FMT administration. However, FMT via colonoscopy is considered the preferred method with high efficacy and safety. The rate of severe complications like aspiration pneumonia after upper gut FMT administration (e.g., duodenal infusion) is considered higher. [70, 77] Techniques using FMT in capsule form deserve attention, as it is more accessible than classical endoscopic invasive procedures [78].

Recently published prospective, single-arm, phase 2a study evaluating the use of pooled allogeneic fecal microbiota (MaaT013) in 76 patients with grade III-IV SR-GI-aGvHD (24 patients - clinical trial NCT03359980 HERACLES study and 52 patents - compassionate use/expanded access program - EAP in France) is the first completed and the most extensive study in the field. Moreover, the microbiota product was obtained as a multi-donor material. The ORR of the gastrointestinal tract (GI-ORR) on day 28 was 38% in the prospective population, and the GI-ORR was 58% in the EAP group. Interestingly, responders on 28 day presented a higher microbiota richness and increased levels of commensal bacteria in the stool, than non-responders. Most bacteria representatives were butyrate producers, considered potentially beneficial in patients with SR-GI-aGvHD [79]. Another single-arm, non-randomized phase III clinical study is ongoing in patients with steroid-resistant or ruxolitinib-resistant/intolerant aGvHD (NCT04769895), which is supposed to be completed in September 2024.

### Cellular therapies

Taking into account the complex pathogenesis of GvHD, there is space for potential roles for adoptive cellular therapies, such as regulatory T cells (Tregs), mesenchymal stromal cells (MSCs), and placenta-derived decidua stromal cells (DSCs) [80].

Preliminary data on cell therapy use in SR-aGvHD patients vary greatly, with complete response rates differing from 13 to 70% [81]. The feasibility and tolerability of case reports and early-phase studies are encouraging. It is necessary to prove the effectiveness of these approaches in randomized clinical studies.

Although there are many ongoing clinical trials of cell therapies worldwide, the broader use of these approaches still needs to be improved in everyday clinical practice. They are primarily developed in academic studies; however, some commercial products are already off the shelf. More research, ideally randomized, is required to define the optimal dosing schedule to validate the clinical efficacy of cellular products, which might be potentially combined with other agents, i.e., with ECP (NCT05333029) or ruxolitinib (NCT04744116) [81].

### Glucagon-like peptide 2 analogs

The gut tissue-protective strategies are considered future targets of GvHD therapy. Glucagon-like peptide 2 (GLP-2), a product of intestinal L cells, acts as an enteroendocrine hormone. In preclinical trials GLP-2 analogues have demonstrated the regenerative potential of intestinal stem cells and Paneth cells, damaged in tissues involved by GI-GvHD [82, 83]. GLP-2 targeted GvHD treatment may rebuild intestinal homeostasis due to the regeneration of intestinal cells. Teduglutide, a human recombinant GLP-2 analogue was reported as a promising agent in managing GI-aGvHD in pediatric patients [84]. Those findings are evaluated in ongoing clinical trial i.e. combining ruxolitinib with apraglutide, a GLP-2 analogue (trial registered as NCT05415410).

### The supportive care – nutrition, supplementation

The nutritional status of patients undergoing transplantation should be regularly assessed using standardized tools to estimate the risk for pre-existing malnutrition [85]. Malnutrition and severe weight loss are commonly reported in allo-HSCT recipients and are associated with increased transplant-related mortality [86, 87]. Gastrointestinal symptoms, such as nausea, vomiting, and diarrhea, significantly impair nutritional status by reducing oral intake, leading to weight and protein loss. Hypoalbuminemia, a feasible malnutrition indicator, has been described as a predictor of worse outcomes for allo-HSCT recipients and a marker for poor response in patients with a steroid-resistant form of GvHD [88–90].

Despite the use of sophisticated GvHD prophylaxis methods during the peri-transplant period, maintaining adequate nutrition is challenging in the early period after allo-HSCT. A traditional neutropenic diet was designed in the 1980s to minimize the risk of infections by reducing exposure to harmful bacteria in raw food. Reports from HSCT centers confirm that a low-microbial diet is preferable during the neutropenic phase [91–93]. A restricted diet might not provide the appropriate calorie intake and be sufficiently nutritious. Recent randomized trials found no significant difference in infection rates between patients on a restrictive neutropenic diet and those on a non-restrictive diet, suggesting that strict dietary limitations may not provide the expected protective benefit. No data confirms the advantages of a neutropenic diet [94]. Safe food handling with strict hand hygiene is recommended to prevent foodborne infections [85].

Patients with peri-transplant complications may require parenteral nutrition (PN) or/and enteral nutrition (EN), which, in long-term dependence, might cause or exacerbate metabolic disorders. Issues such as hyperglycemia, electrolyte disorders, and fluid imbalance are already present in patients suffering from GvHD affecting the gut. Considering the physiological and nutritional route, an oral diet with EN is preferable to support the nutritional status and protect the mucosal gut barrier. However, this might not be feasible for patients with severe GI-aGvHD who might require PN [95]. Ongoing attempts are to validate dietary interventions as models for clinical trials in GI-aGvHD, though data in the field are limited [96, 97].

The use of nutritional supplements in HSCT survivors, particularly GvHD patients, may be controversial [100]. Vitamin D supplementation is recommended, as beneficial routine supplementation was confirmed [98]. In the context of GI-aGvHD patients, strategies supportive of gut microbiota have been evaluated. Implementing safe high-fiber products might contribute to producing short-chain fatty acids, supporting the intestinal microbiota and immunomodulatory mechanisms, potentially preventing the development of GI-GvHD, and improving outcomes [99]. Limpert et al. [100] reviewed promising results from preclinical and clinical trials using dietary and nutritional interventions, including amino acids, oligosaccharides, and prebiotics, as therapeutic approaches in aGvHD targeting the gut microbiota. However, the potentially beneficial impact has to be explored in randomized controlled trials. Optimizing nutrition in allo-HSCT recipients, particularly those with GvHD, is critical yet challenging. Future research should focus on validating these approaches in clinical trials to establish evidence-based guidelines for nutritional support in this vulnerable population.

### Summary

Indeed, prophylactic and therapeutic strategies for aGvHD have significantly improved worldwide over the past few years. The GvHD prophylaxis with PTCy, which has been used in haploidentical settings, also applies in classical allogeneic approaches. The second-line therapy for aGvHD has been established and is available in most of the transplant centers. However, there are still challenges in aGvHD. Considering the risk of infections and relapse, the most effective and safe prevention strategies remain to define. Steroids have been the first-line treatment for many years, with a response rate amounting to 50% and associated complications. Therefore, the strategy combined with steroids might be implemented earlier based on individual risk. The development of risk stratification tools remains a field of interest and may lead to risk-based treatment. Despite developing risk stratification models using biomarkers, they still need to be used in everyday practice. The lack of cost-effective tools, which might help to stratify patients, limits therapy adjustment dedicated to the patient. Moreover, comparing clinical trials remains challenging. Because the risk stratification of study subjects are not homogenous. The majority of studies include newly diagnosed aGvHD patients either with low-risk aGVHD, skin-limited manifestation, or lower grade of severity than III-IV grade in gastrointestinal involvement. Patients with steroid-resistant disease have often presented metabolic complications already and are at higher risk of infectious complications and cytopenia, which might be life-threatening. The management of severe cases, in particular steroid and ruxolitinib-resistant aGvHD patients, remains an unmet medical need and requires modern approaches available in clinical practice. Strategies targeting gut microbiota, i.e., fecal microbiota intervention, gut tissue-protective therapies, and nutritional interventions, seem promising and safe but still need further investigation.

## Data Availability

No datasets were generated or analysed during the current study.
